# Dementia risk prediction in individuals with mild cognitive impairment: a comparison of Cox regression and machine learning models

**DOI:** 10.1186/s12874-022-01754-y

**Published:** 2022-11-02

**Authors:** Meng Wang, Matthew Greenberg, Nils D. Forkert, Thierry Chekouo, Gabriel Afriyie, Zahinoor Ismail, Eric E. Smith, Tolulope T. Sajobi

**Affiliations:** 1 Department of Community Health Sciences, Cumming School of Medicine, University of Calgary, 3280 Hospital Drive NW, T2N 4Z6 AB Calgary, Canada; 2 Department of Clinical Neurosciences & Hotchkiss Brain Institute, Cumming School of Medicine, University of Calgary, Calgary, AB Canada; 3 Department of Mathematics and Statistics, University of Calgary, Calgary, Canada; 4 Department of Radiology, Cumming School of Medicine, University of Calgary, Calgary, AB Canada; 5 Division of Biostatistics, School of Public Health, University of Minnesota, Minneapolis, US; 6 Department of Psychiatry, Cumming School of Medicine, University of Calgary, Calgary, Canada

**Keywords:** Time-to-event outcomes, Dementia, Risk prediction, Cox regression, Machine learning

## Abstract

**Background:**

Cox proportional hazards regression models and machine learning models are widely used for predicting the risk of dementia. Existing comparisons of these models have mostly been based on empirical datasets and have yielded mixed results. This study examines the accuracy of various machine learning and of the Cox regression models for predicting time-to-event outcomes using Monte Carlo simulation in people with mild cognitive impairment (MCI).

**Methods:**

The predictive accuracy of nine time-to-event regression and machine learning models were investigated. These models include Cox regression, penalized Cox regression (with Ridge, LASSO, and elastic net penalties), survival trees, random survival forests, survival support vector machines, artificial neural networks, and extreme gradient boosting. Simulation data were generated using study design and data characteristics of a clinical registry and a large community-based registry of patients with MCI. The predictive performance of these models was evaluated based on three-fold cross-validation via Harrell’s concordance index (c-index), integrated calibration index (ICI), and integrated brier score (IBS).

**Results:**

Cox regression and machine learning model had comparable predictive accuracy across three different performance metrics and data-analytic conditions. The estimated c-index values for Cox regression, random survival forests, and extreme gradient boosting were 0.70, 0.69 and 0.70, respectively, when the data were generated from a Cox regression model in a large sample-size conditions. In contrast, the estimated c-index values for these models were 0.64, 0.64, and 0.65 when the data were generated from a random survival forest in a large sample size conditions. Both Cox regression and random survival forest had the lowest ICI values (0.12 for a large sample size and 0.18 for a small sample size) among all the investigated models regardless of sample size and data generating model.

**Conclusion:**

Cox regression models have comparable, and sometimes better predictive performance, than more complex machine learning models. We recommend that the choice among these models should be guided by important considerations for research hypotheses, model interpretability, and type of data.

**Supplementary Information:**

The online version contains supplementary material available at 10.1186/s12874-022-01754-y.

## Introduction

Dementia is a complex health condition influencing memory, thinking, behavior, and quality of life. In 2015, the worldwide costs of dementia were estimated at $818 billion USD and 86% of the costs were incurred in high-income countries [1]. Dementia is usually preceded by mild cognitive impairment (MCI), defined as cognitive concerns with poor cognitive test scores despite preserved activities of daily living. Individuals with MCI have a substantially higher risk of developing dementia compared to people with normal cognition. On average, 5-10% of people with MCI progress to dementia per year [2]; however, not all individuals with MCI progress to dementia [3]. In the absence of disease-modifying treatments for dementia, there is increased demand for clinical decision aids to support early identification of individuals with high risk of developing dementia and who may benefit from target interventions for modifiable risk factors. Such tools can also be used for surveillance purposes: when individuals with MCI progress to dementia, they can receive supportive care to live safely in the community [4]. In addition, such tools can support care providers in answering questions for patients with MCI and their families about the risk of developing dementia and future life planning [5].

Prognostic risk prediction models [6], which estimates the probability of developing dementia based on a set of patients’ risk factors, constitute a class of models on which such decision tools can be developed. Prognostic risk scores for dementia have been developed based on regression analysis and machine learning models, with the latter being frequently used in recent years. For example, a recently published systematic review of dementia risk scores showed that about 40% of the published models adopted a machine learning algorithm [7]. However, the review concluded that most of the identified risk scores have inherent methodological limitations, which include the lack of internal and external validations of the models, choice of statistical methods for developing the risk scores, and the long interval elapsed between assessments of individuals at risk [7]. To date, no suitable dementia risk score have been adopted as a clinical decision aid for use in routine clinical practice [7, 8].

The increased uptake of machine learning models in clinical research is predicated the assumption that ML is inherently more powerful than traditional regression models because these are non-linear models that can capture non-linear associations between explanatory variables and the outcome of interest. However, previous studies reported no significant differences in the model performance between machine learning and logistic regression models from a systematic review of empirical studies [9]. Such comparisons limit the external generalizability of conclusions about the performances of the models. Monte Carlo simulation methodology is an alternative approach for assessing comparability of several models under a variety of data analytic conditions[10]. Although there are a few simulation studies that compared statistical and machine learning approaches to risk prediction for time-to-event data [11], these simulation studies are often biased towards the novel approach. There is need for unbiased comparisons of machine learning and traditional statistical methods to guide the selection of optimal methodology for developing clinical risk prediction for dementia [11]. The overarching aim of this study is to investigate the comparative performance of machine learning and (unpenalized and penalized) Cox regression models for predicting time-to-event outcomes (MCI to dementia progression) under a variety of data-analytic conditions. We hypothesize that Cox regression will have comparable and/or even better accuracy (with respect to discrimination and calibration) to machine learning algorithms.

## Methods

To ensure that the design of the simulation and simulation conditions investigated reflect the characteristics and features of real-world datasets, data from a clinical registry and a large community-based registry of people with MCI were used to inform the selection of simulation conditions.

### Prospective Registry for persons with memory symptoms (PROMPT) Registry

The PROMPT registry of the Cognitive Neurosciences Clinic at the University of Calgary is a memory clinic registry enrolling all patients with MCI. The PROMPT Registry was established in July 2010 and enrolls patients referred to the Cognitive Neuroscience Clinic in Calgary, Alberta, Canada, for assessing suspected impairment in cognitive or behavioral function [12]. All patients attending the clinic are eligible to participate. To ensure complete follow-up, we linked PROMPT participants to Alberta healthcare administrative data for surveillance of new dementia diagnoses. There were 273 patients with MCI in PROMPT (up until April 2020), with age 55 years and older, with data on baseline predictors, and with at least one follow-up after the baseline visit. The study predictors include demographic characteristics (age, sex, education, marital status, first language, and handedness), cognitive tests or profiles (total score for the Consortium to Establish a Registry for Alzheimer’s Disease neuropsychological battery, the Montreal Cognitive Assessment [MoCA] total score, mild behaviour impairment total score, any neurological signs, family history, and cognitive complaints from informants), life-style factors (smoking and alcohol abuse), as well as health history (hypertension, dyslipidemia, diabetes, hypothyroidism, traumatic brain injury, cerebrovascular disease, cardiovascular disease, mood disorders, insomnia, obstructive sleep apnea, any neurological disorders, and psychiatric diseases other than mood disorder).

### National Alzheimer’s Coordinating Center’s (NACC) Registry

The NACC was established by the National Institute on Aging (NIA)-funded Alzheimer’s Disease Research Centers (ADRCs) that recruits and collects data on subjects with cognitive function ranging from normal to dementia. The NACC cohort used in this study are from Uniform Data Set (NACC-UDS; naccdata.org). The NACC-UDS is a longitudinal dataset that includes demographic and standardized clinical data collected approximately annually. All test centers administered standardized forms. Detailed information on the cohort and the neuropsychological battery of tests included in the UDS are described elsewhere [13–15]. Patient data were prospectively collected on pre-specified case report forms. We used version 3 (refers to those enrolled from 2015 onwards) of the NACC due to significant changes from previous versions. There were 967 patients with MCI in NACC, with age 55 years and older, with data on baseline predictors, and with at least one follow-up after the baseline visit. The identification of these two study cohorts can be found in our Support Document (Figure S1-S2). The study predictors include demographic characteristics (age, sex, education, race, first language, handedness, and marital status), cognitive tests or profiles (time since initial cognitive decline, memory complaints from subjects or informants, family history, MoCA total score, geriatric depression scale, neuropsychiatric symptoms total score, clinician diagnosed behavioural symptoms, motor symptoms, and overall course of the decline), life-style factors (smoking), whether referred by health professionals, primary reason for coming to Alzheimer’s disease centers, as well as medical history (hypertension, diabetes, hypercholesterolemia, arthritis, urinary incontinence, cerebrovascular disease, cardiovascular disease, rapid eye movement sleep behaviour disorder, insomnia, traumatic brain injury, cancer, use of nonsteroidal anti-inflammatory medication, thyroid disease, mood disorder, parkinsonism, and Vitamine B12 deficiency), and physical exams (body mass index, vision, and hearing).

These two registries were selected for informing the design of this simulation study because of their different designs (i.e., sample size, type of patient population, number of predictors) and data characteristics (i.e., time-to-event distribution and censoring rate), which could ensure that our simulation and the conditions investigated mimic real-world studies.

For both cohorts, the survival outcome is the time to all-cause dementia over a three-year period following MCI diagnosis which is based on the standard outcome definitions, including Diagnostic and Statistical Manual of Mental Disorders and the National Institute on Aging – Alzheimer’s Association [16, 17]. In the PROMPT (N = 273) and NACC (N = 967) registries, 110 (40%) and 224 (23%) of individuals developed dementia from MCI at baseline within three years, respectively. Candidate predictors were all measured at baseline in both cohorts. We acknowledge that people with MCI may never develop dementia [3], in this current study, individuals who did not develop dementia during the three-year period following MCI diagnosis were assumed to be right-censored. Descriptive analysis of the sample characteristics was included in our Support document Table S1-S2.

### Simulation study

The prediction models compared in this simulation study including (1) Cox regression, (2) Cox regression with least absolute shrinkage selection operator (LASSO-Cox), (3) Cox regression with ridge penalty (Ridge-Cox), (4) Cox regression with elastic net (EN-Cox), (5) survival trees, (6) random survival forests, (7) survival support vector machines, (8) survival neural networks, and (9) extreme gradient boosting.

A survival tree refers to a non-parametric time-to-event regression model in which the sample is recursively partitioned into homogeneous subgroups based on input predictors that maximizes the differences in survival distributions for the subgroups [18]. Random survival forests [19] is a direct extension of the random forests [20] to handle time-to-event outcomes. Random survival forests [19] function the same as conventional random forests models, except that each splitting is optimized by maximizing survival differences between daughter nodes. The outcome of random survival forests is the ensemble estimate for the cumulative hazard function. Survival support vector machines have been extended to analyze censored data including regression [21], ranking [22], and hybrid approach [23]. The regression method handles survival times as a standard regression problem (without penalization for censored observations). The ranking approach is essentially treating the support vector machine-based survival analysis as a classification problem with an ordinal target variable. The hybrid approach combines the regression and ranking methods. Since it was previously reported that regression constraints perform significantly better than ranking constraints, and similar to the performance of the hybrid approach on clinical data [23], we adopted the regression approach in this work. The survival deep neural network “DeepSurv” [24], is a non-linear Cox proportional hazard deep feed-forward neural network that can model hazard rates. Specifically, the patients’ baseline data serves as the input to the first layer, which is then processed and combined in the hidden layers followed by a dropout layer to reduce overfitting [25]. The output layer produces estimates for the hazard rates similar to Cox regression model. The corresponding cost function combines the Cox partial likelihood with regularization, and gradient descent optimization is used for estimating the parameters. The extreme gradient boosting algorithm is an effective and flexible machine learning method [26], which extends the original gradient boosting machine [27] with flexibilities for different types of outcomes (e.g., binary, continuous, or survival data). The extreme gradient boosting survival model can be used to process the right-censored survival data; it relaxes the linearity assumption between log-hazard ratio and covariates and still has proportional hazards assumption [28, 29].

#### Simulation conditions

The simulation conditions to be investigated include (a) sample size (*N*), (b) censoring rate (*CR*), (c) the data generating processes (the relationship between the predictors and hazard function), and (d) number of predictors. In order to ensure external generalizability of the study results, the data-analytic conditions investigated in our simulation study were selected to mimic real-world data scenarios obtainable in existing registries [30, 31]. We simulated data based on the covariates (design matrix) of two cohorts of patients with MCI. With each cohort, two different data generating processes based on fitting either Cox regression (a typical conventional regression) or random survival forests (a typical machine learning exploring non-linearity and high-order interactions) were used. Simulated time-to-event outcomes were then generated based on predicted survival distribution and predicted failure probability (MCI to dementia conversion) at three years.

**Fig. 1:**
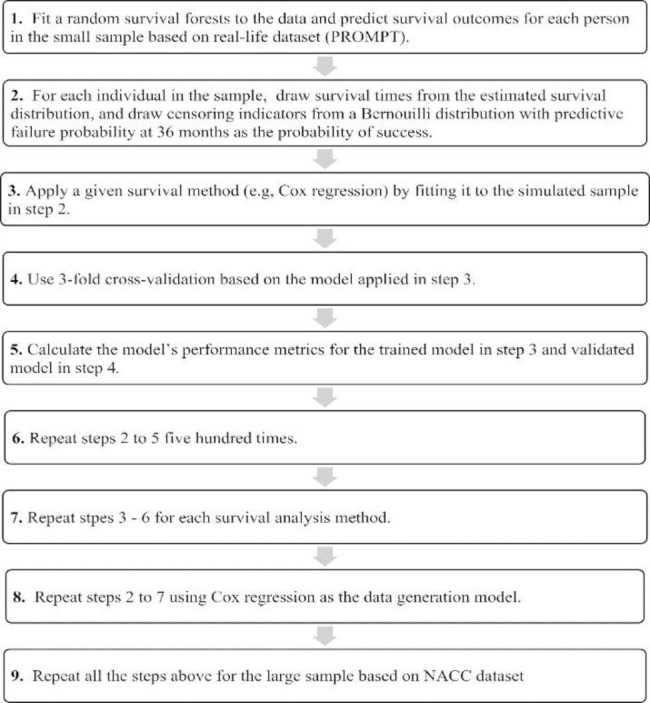
The process of simulation was conducted as follows:

The data generation process is described as shown in Fig. 1. Cox regression model and random survival forests were used to fit and predict survival outcomes (survival distribution and failure probability at three-years) for each individual in the real-life sample. The study outcome data were no longer used after this step. The model (either Cox regression or random survival forests) predicted the survival distribution $\widehat{{S}_{i}\left(t\right)}$ for each individual representing the estimates of the random variable survival time $t$ for an individual $i$ observation. Next, a random simulation function [32] was used to draw simulations (i.e., survival times) from the distribution for each individual in the sample. The censoring indicator was simulated using a Bernoulli distribution with the given individual-specific probability $\widehat{{p}_{i}}$, which was the predicted failure probability at three-years based on either Cox regression or random survival forests. The simulation data were generated based on Cox regression models and random survival forests also because they had relatively better model performance compared with the rest of the compared methods (details are in our support document Table S3).

The performance of each model was evaluated using measures of discrimination and calibration based on three-fold cross-validation (CV). Specifically, Harrell’s concordance index (c-index) [33], integrated calibration index (ICI) for survival models [34], and integrated brier score (IBS) [35] were used to assess model accuracy. The c-index allows for the computation of concordance probability, measuring the proportion of the pairs (those can be compared) where the observation with the higher survival time has the higher predicted survival probability by the model.

A numerical calibration metric ICI [34] has been extended to evaluate the calibration of predicted probabilities for survival models and it is estimated as the mean absolute differences between observed and predicted probabilities across the sample.

$$ICI=\frac{1}{n} {\sum }_{k=1}^{n}\left|\widehat{{p}_{{t}^{*}}^{ \left(k\right)c }}- \widehat{{ p}_{{t}^{*}}^{\left(k\right)}}\right|$$

$$\widehat{{p }_{{t}^{*}}^{\left(k\right)}}=F \left({t}^{*} \right|\varvec{X})$$

$$\text{log}(-\text{log}(1-\widehat{{p}_{{t}^{*}}^{ \left(k\right)c }}))=g (\text{log}\left(-\text{log}\left(1-\widehat{{ p}_{{t}^{*}}^{\left(k\right)}}\right)\right), t)$$

Let $F \left({t}^{*} \right|\varvec{X})$ denote a survival model for estimating the failure probability (e.g., the probability of developing dementia) for a subject with covariate vector $\mathbf{X}$; $\widehat{{p }_{{t}^{*}}^{\left(k\right)}}$refers to the ${\text{k}}^{\text{t}\text{h}}$ predicted failure probability before time ${t}^{*}$ based on the fitted model $F \left({t}^{*} \right|\varvec{X})$; $n$ denotes the number of unique predictions from the fitted model; $\widehat{{ p}_{{t}^{*}}^{ \left(k\right)c }}$refers to estimated observed failure probability corresponding to the given predicted probability

$$\widehat{{ p}_{{t}^{*}}^{\left(k\right)}}$$

based on the relationship between

$$\text{log}\left(-\text{log}\left(1-\widehat{{ p}_{{t}^{*}}^{\left(k\right)}}\right)\right)$$

and the

$$\text{log}\left(-\text{log}\left(1-\widehat{{p}_{{t}^{*}}^{ \left(k\right)c }}\right)\right)$$

function g, which can be estimated using either a flexible adaptive hazard regression [36] or using a Cox regression model with restricted cubic splines [34]. The latter method with three knots was used in this work since it was demonstrated that the two approaches had comparable performance [34]. A smaller ICI value indicates a better calibrated prediction model and this metric is suggested to use in comparing different prediction models [34].

The IBS, which uses a squared loss function [35], is defined as

$$L\left(S\right)=\frac{1}{NT} \sum _{i=1}^{N}\sum _{j=1}^{T}{L \left({S}_{i},{t}_{i} \right|}^{ }{t}_{j}^{*})$$.

Here, an approximation to integration is made by taking the sample mean over all $T$ unique time points and all $N$ observations, where $N$ is the number of observations, ${S}_{i}$ is the predicted survival function for individual $i$, and ${t}_{i}$is the survival time. For an individual who developed dementia at time $t$, with predicted survival function, S, at time ${t}^{*}$, is defined by:

$$\eqalign{ {L \left(S,t \right|t}^{*})=\frac{S{\left({t}^{*}\right)}^{2} I (t\le {t}^{*}, \delta =1)}{G\left(t\right)}+ \\ \frac{(1-S{\left({t}^{*}\right))}^{2} I (t>{t}^{*})}{G\left(t\right)}}$$,

where $G$ is the Kaplan-Meier estimate of the censoring distribution. $I \left(\bullet \right)$ is the indicator function for example,

$$\eqalign{ I \left(t\le {t}^{*}, \delta =1\right)=1 \\ if \ \ t\le {t}^{*} \ \ and \ \ delta =1;\\otherwise \ \ I \left(t\le {t}^{*}, \delta =1\right)=0}$$

A smaller IBS value indicates better calibration and shows better combination of discrimination and calibration.

Hyper-parameter tuning was performed for each survival method, except for unpenalized Cox regression, based on random grid search (1000 evaluations in total) using five-fold CV with c-index as the scoring metric. The search space for each hyper-parameter and the tuned hyper-parameters used are included in our support document (Table S4). For each combination of simulation condition, a total of 500 replications was drawn based on each data generation process. All analyses were conducted using the R statistical programming language [37].

## Simulation results

### Model performance measure: c-index

**Fig. 2:**
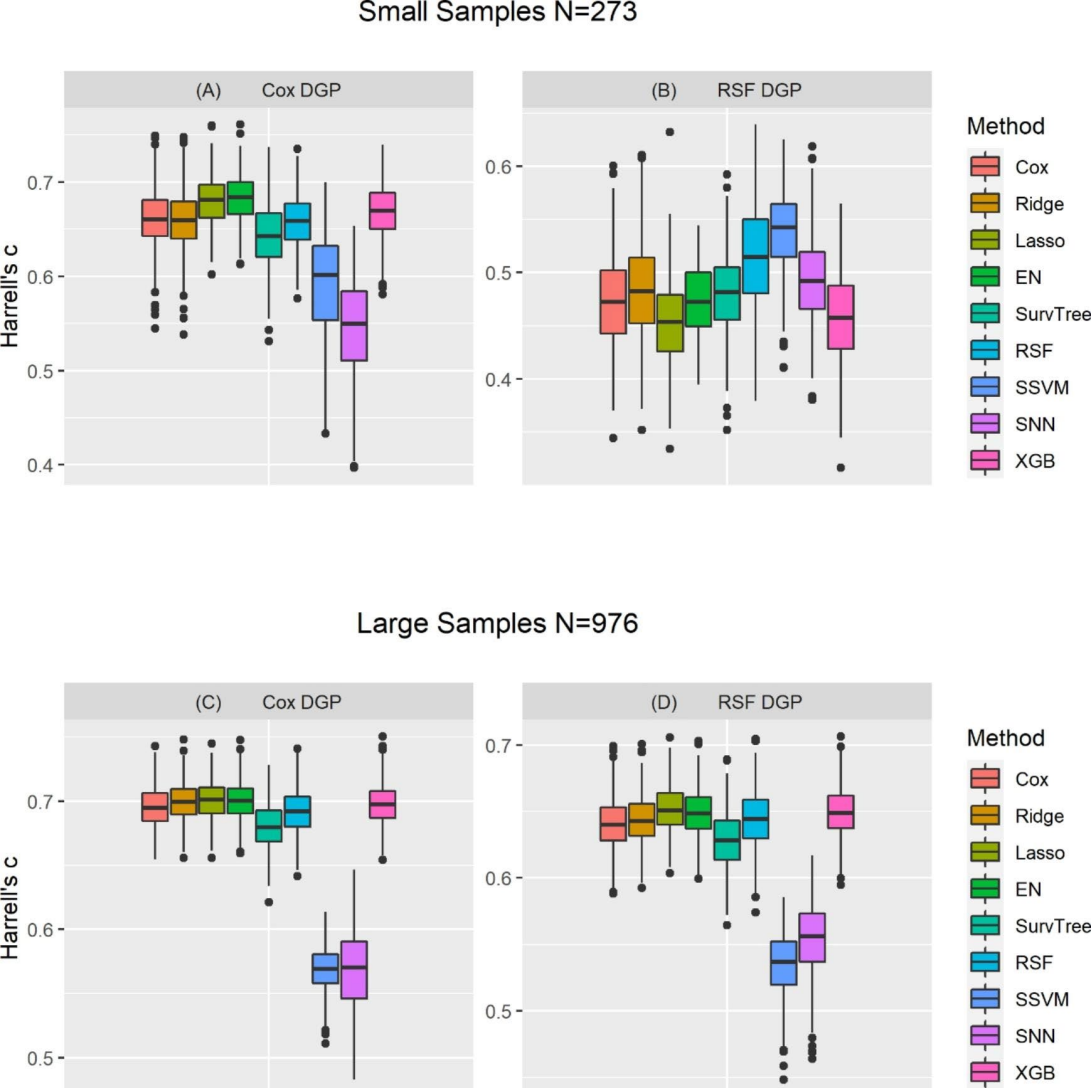
Distribution of the estimated c-index of nine models, assessed from three-fold CV across 500 replications NB: There are four panels, with the top two panels (A and B) are for the small samples (based on characteristics and features of PROMPT dataset), the bottom two panels (C and D) are for the large samples (based on characteristics and features of NACC dataset). Left and right panel are for the Cox regression used for data generating process [DGP] and random survival forests [RSF] based DGP, respectively. Each panel consists of nine boxplots corresponding to each of the nine survival analysis models. Each boxplot shows the variation in the Harrell’s c-index [c-index] across the 500 simulation replicates when a certain DGP and survival analysis method were applied. Cox: Cox proportional hazards; Ridge-Cox: Cox regression based on ridge penalty; LASSO-Cox: Cox regression based on Least Absolute Shrinkage Selection Operator penalty; EN-Cox: Cox regression based on elastic net penalty; SurvTree: Survival Tree; RSF: Random survival forests; SSVM: Survival support vector machine; SNN: Survival neural networks; XGBoost: Extreme gradient boosting

Figure 2 describes the distribution of accuracy for the nine survival models using Harrell’s c-index for the simulation conditions when the data generating process was based on Cox regression and random survival forests. Simulated conditions represented relatively small sample size (based on characteristics and features of PROMPT dataset) and relatively large sample (based on characteristics and features of NACC dataset). When the data were generated based on a Cox regression model, the estimated c-index values for all the models were generally higher than when the data were simulated based on random survival forests, regardless of the sample size.

When the data were generated based on small sample size and Cox regression, the estimated mean c-index was the highest for penalized Cox regression with elastic net (EN-Cox) and with LASSO (LASSO-Cox), but the lowest for survival neural networks. In addition, the estimated mean c-index values for random survival forests, Cox regression, and Ridge-Cox were slightly lower than the EN-Cox and LASSO-Cox. On the other hand, when data were generated based on a random survival forests model, only random survival forests and survival support vector machines had estimated mean c-index values greater than 0.5, The estimated mean c-index values for Cox regression, Ridge-Cox, EN-Cox, survival trees, and survival neural networks were comparable but less than 0.5. When the data were generated based on a large sample size, regardless of the data generation model, there were negligible differences between the estimated c-index values for most of the models, except for survival support vector machine and survival neural networks models, which had the lowest estimated c-index values.

### Model performance measure: calibration

**Fig. 3:**
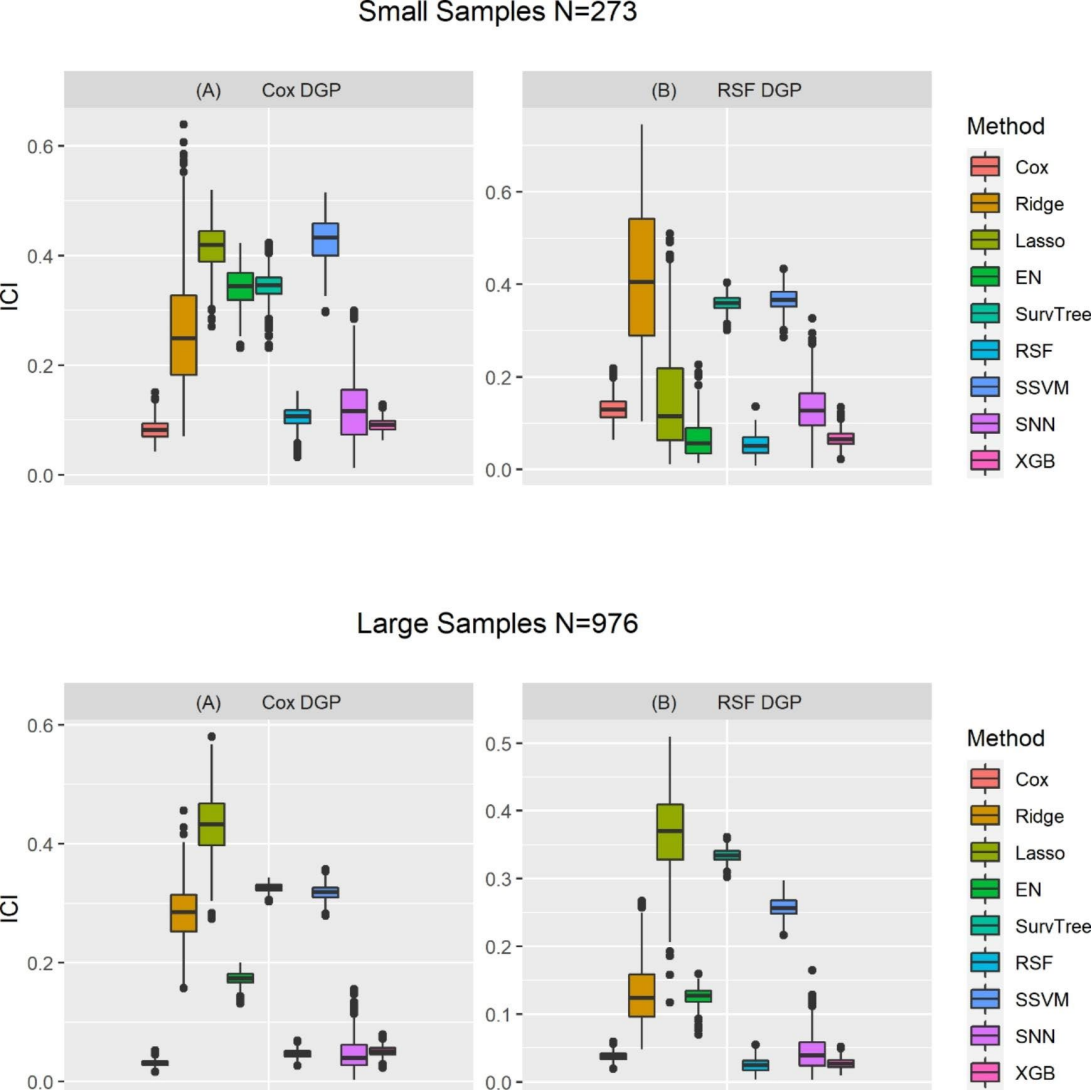
Distribution of the estimated ICI of nine models, assessed from three-fold CV across 500 replications NB: There are four panels, with the top two panels (A and B) are for the small samples (based on characteristics and features of PROMPT dataset), the bottom two panels (C and D) are for the large samples (based on characteristics and features of NACC dataset). Left and right panel are for the Cox regression used for data generating process [DGP] and random survival forests [RSF] based DGP, respectively. Each panel consists of nine boxplots corresponding to each of the nine survival analysis models. Each boxplot shows the variation in the integrated calibration index [ICI] across the 500 simulation replicates when a certain DGP and survival analysis method were applied). Cox: Cox proportional hazards; Ridge-Cox: Cox regression based on ridge penalty; LASSO-Cox: Cox regression based on Least Absolute Shrinkage Selection Operator penalty; EN-Cox: Cox regression based on elastic net penalty; SurvTree: Survival Tree; RSF: Random survival forests; SSVM: Survival support vector machine; SNN: Survival neural networks; XGBoost: Extreme gradient boosting

Figure 3 describes the distribution of ICI values for all nine models, out of 500 replications for Cox regression and random survival forests data generation processes. When the sample size was small and Cox regression model was used to generate time-to-event outcomes, the extreme gradient boosting, random survival forest, survival neural networks, and Cox regression had the lowest estimated ICI values. In addition, the LASSO-Cox and survival support vector machine model had higher estimated ICI values, suggesting these two models were more poorly calibrated than the rest of the models. On the other hand, when the simulation data were generated based on random survival forests, the extreme gradient boosting, EN-Cox, and random survival forest tended to result in estimates with the lowest ICI values in the simulation replications. The Cox regression model, LASSO-Cox, and survival neural networks had slightly higher estimated ICI values than random survival forests. Furthermore, when sample size was large, regardless of the data generation model, the extreme gradient boosting, random survival forest, survival neural networks, and Cox regression tended to result in estimates with the lowest ICI values in the simulation replications than the rest of the models. On the contrary, LASSO-Cox tended to result in estimates with the highest ICI values compared with the remaining models.

**Fig. 4:**
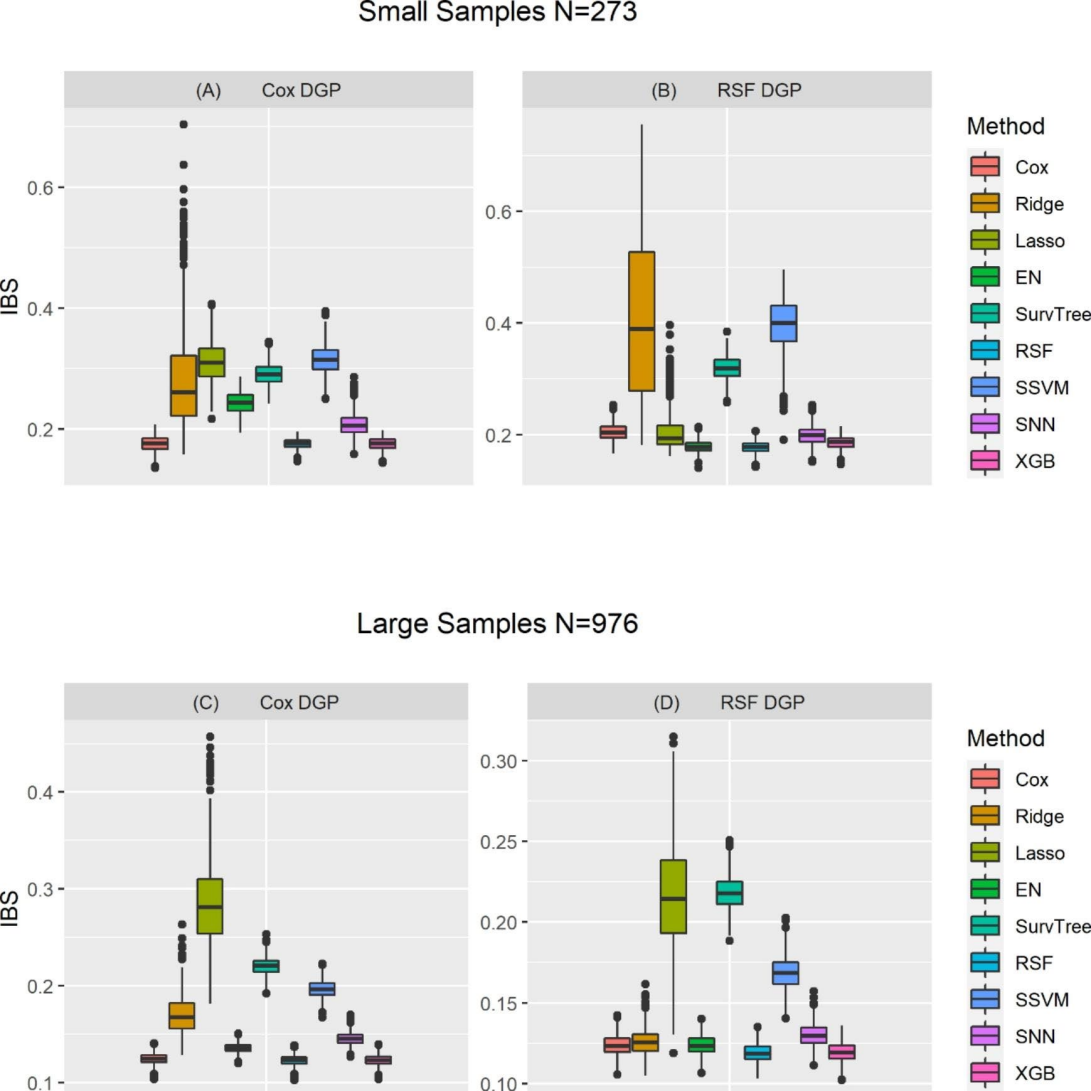
Distribution of the estimated IBS of nine models, assessed from three-fold CV across 500 replications NB: There are four panels, with the top two panels (A and B) are for the small samples (based on characteristics and features of PROMPT dataset), the bottom two panels (C and D) are for the large samples (based on characteristics and features of NACC dataset). Left and right panel are for the Cox regression used for data generating process [DGP] and random survival forests [RSF] based DGP, respectively. Each panel consists of nine boxplots corresponding to each of the nine survival analysis models. Each boxplot shows the variation in the integrated brier score [IBS] across the 500 simulation replicates when a certain DGP and survival analysis method were applied). Cox: Cox proportional hazards; Ridge-Cox: Cox regression based on ridge penalty; LASSO-Cox: Cox regression based on Least Absolute Shrinkage Selection Operator penalty; EN-Cox: Cox regression based on elastic net penalty; SurvTree: Survival Tree; RSF: Random survival forests; SSVM: Survival support vector machine; SNN: Survival neural networks; XGBoost: Extreme gradient boosting

Figure 4 describes the distribution of the IBS values for all the investigated models when the data generation process was based on Cox regression and random survival forests. When data were generated based on Cox regression for small sample size condition, random survival forests, extreme gradient boosting, and Cox regression model had lower estimated mean IBS values. In contrast, LASSO-Cox and the survival support vector machine models had the highest estimated mean IBS values. When the simulation data was generated based on random survival forests model, Cox, LASSO-Cox, EN-Cox, survival neural networks, extreme gradient boosting, and random survival forests all had comparable estimated IBS values. On the other hand, Ridge-Cox survival trees, and survival support vector machines had higher estimated mean IBS values than the rest of the models. When sample size was large and the data generating process was based on Cox regression, EN-Cox, random survival forests, survival neural networks, and extreme gradient boosting all had analogous estimated IBS values as Cox regression model. However, LASSO-Cox had the highest estimated mean IBS value. In contrast, when the data were generated based on random survival forests, the Cox regression, Ridge-Cox, EN-Cox, random survival forests, survival neural networks, and extreme gradient boosting all had relatively small and similar estimated IBS values, while the LASSO-Cox and survival trees had higher estimated IBS values.

## Discussion

This study evaluated the accuracy of (penalized and unpenalized) Cox regression and machine learning survival models using Monte Carlo simulation. Our simulation results show that Cox regression and machine learning model had comparable predictive accuracy across three different performance metrics and data-analytic conditions. Unpenalized Cox regression, random survival forests, and extreme gradient boosting had slightly higher estimated discriminatory performance (i.e., c-index) and were better calibrated than the remaining models, regardless of data generation model and sample size.

These findings are consistent with conclusions from previous studies that have investigated the performance of conventional regression models and machine learning models for predicting binary outcomes [9, 30]. In a systematic review of 71 studies that employed logistic regression models and machine learning models for binary outcome predictions, the authors found no significant differences in the discriminatory performance between machine learning and logistic regression models [9]. A recent study used Monte Carlo simulations to compare logistic regression with machine learning models and concluded that these models had comparative performance with respect to predictive accuracy, whereas (penalized) logistic regression and boosted trees tended to have better performance compared to other methods [30].

A major implication of our study conclusion is that the choice between Cox regression and machine learning algorithms for predicting time-to-event outcomes should be based on important considerations other than model accuracy since there is no significant difference in the discriminatory performance of both classes of models. Considerations for research objectives and data characteristics (e.g., outcome distribution, type, and number of predictors), and interpretability of the model results could influence model selections. For example, Cox regression models could be more suitable for addressing research questions when quantifying and explaining the impact of predictors on time-to-event outcomes are of interests. In contrast, machine learning algorithms might be more suited for prediction in datasets that include unconventional predictor variables, such as voxel-level imaging data.

This study has several strengths and some limitations. A strength of this study is that the design and implementation of the simulation study were informed by real-world data characteristics rather than theoretical distributional data characteristics which might be unrealistic in data analytic conditions encountered in dementia research. Second, a more robust approach to the design and implementation of the simulation study was taken to ensure unbiased comparison of the predictive performance of the models investigated. The simulation study was comprehensive in its investigation of both discrimination and calibration for the model performance and use of multiple data generation models that ensure the fairness of the model comparison. Nevertheless, this study has limitations. First, the simulation conditions investigated were limited to data characteristics (e.g., between-predictor correlation structures) commonly seen in existing registries and observational studies of patients with mild cognitive disorders and not representative of the general population. While our simulation conditions did not examine the data characteristics seen in other populations (e.g., cardiac population investigated by [30]), our findings are consistent with conclusions from other relevant simulation studies in other patient populations [30]. Second, our simulation study assumed that there was no competing event that could have prevented the occurrence of dementia during the three-year period. Competing events, such as mortality, can mask the observation of progression to dementia, especially in the population of MCI patients which is predominantly made up to older adults and the elderly. Future research will explore the impact of competing risk of death on the accuracy of these models. Another limitation is the significant drop in predictive accuracy of all nine models when the simulation data were generated based on the random survival forests in small samples (N = 276), although this was not the case when Cox regression was used as the data generating model. This decrease in predictive accuracy could be attributed to the fact that the random survival forests data generation process is sensitive to the small sample size of the PROMPT registry. Finally, our simulation study assumed that the study predictors included in the model were known and hyper-parameter tuning was used to reduce model overfitting. Future studies will explore the impact of variable selection methods on the accuracy of (penalized and unpenalized) Cox regression and machine learning models.

## Conclusion

In summary, machine learning and traditional regression models remain widely used methodologies for developing clinical risk predictions models. Our study reveals negligible differences in the discrimination and calibration of Cox regression and machine learning models, such as survival random forest, for predicting time-to-event outcomes. This study adds to the body of literature investigating the comparative performance of these two classes of models, which are widely used in prognostic research. Since conventional Cox regression model are generally more interpretable and are well-calibrated than most machine learning algorithms, we recommend its uptake for developing clinical risk prediction models. More importantly, the choice among these models should be guided by important considerations for research hypotheses, model interpretability, and type of data. Future studies will investigate how the variable selection methods influence the accuracy of these models.

## Electronic supplementary material

Below is the link to the electronic supplementary material.

Supplementary Material 1

## Data Availability

The data that support the findings of this study are available from the principal investigator (Eric Smith: eesmith@ucalgary.ca) of the PROMPT registry and NACC Uniform Data Set (data request link: https://naccdata.org/requesting-data/submit-data-request) but restrictions apply to the availability of these data, which were used under license for the current study, and so are not publicly available. Data are however available from the authors upon reasonable request and with permission of the principal investigator of the PROMPT registry and the approval of NACC data access.

## List of abbreviations

MCI: mild cognitive impairment
PROMPT: Prospective Registry for Persons with Memory Symptoms Registry
MoCA: the Montreal Cognitive Assessment
NACC: the National Alzheimer’s Coordinating Center’s Registry
NIA: National Institute on Aging
ADRCs: Alzheimer’s Disease Research Centers
UDS: Uniform Data Set
N: sample size
CR: censoring rate
CV: cross-validation
c-index: Harrell’s concordance index
ICI: integrated calibration index
IBS: integrated brier score
DGP: data generating process
Cox: Cox proportional hazards
Ridge-Cox: Cox regression based on ridge penalty
LASSO-Cox: Cox regression based on Least Absolute Shrinkage Selection Operator penalty
EN-Cox: Cox regression based on elastic net penalty
SurvTree: Survival Tree
RSF: Random survival forests
SSVM: Survival support vector machine
SNN: Survival neural networks
XGBoost: Extreme gradient boosting
