# Supplementary material for: Dementia risk prediction in individuals with mild cognitive impairment: a comparison of Cox regression and machine learning models
Source: BMC Med Res Methodol. 2022 Nov 2;22:284. doi: 10.1186/s12874-022-01754-y (PMC9628121; doi:10.1186/s12874-022-01754-y)
Supplement: Supplementary file 1 — Supplementary Material 1 [file 12874_2022_1754_MOESM1_ESM.docx]

# Support Document

**Table S1.** Variable measures in PROMPT

| **Variables** | **N=273** |
| --- | --- |
| **Outcome variables** |  |
| Dementia diagnosis in 3 years, n (%) | 110 (40.3) |
| Event time in months, median (Q1-Q3) | 15.7 [6.3-36.0] |
| **Potential predictors** |  |
| Age, mean (SD) | 67.3 (8.3) |
| Female, n (%) | 114 (41.8) |
| Education in years, mean (SD) | 13.4 (3.6) |
| Married, n (%) | 200 (73.3) |
| Family history, n (%) | 66 (24.2) |
| Right-handed, n (%) | 248 (90.8) |
| English first language, n (%) | 246 (90.1) |
| CERAD total score, mean (SD) | 98.6 (17.0) |
| MoCA, mean (SD) | 21.0 (3.8) |
| MBI total score, mean (SD) | 8.2 (10.0) |
| Cognitive complaints (2+ years) from informant, n (%) | 146 (53.5) |
| Any neurological signs, n (%) | 64 (23.4) |
| Smoked, n (%) | 126 (46.2) |
| Alcohol abuse, n (%) | 43 (15.8) |
| Hypertension, n (%) | 139 (50.9) |
| Dyslipidemia, n (%) | 128 (46.9) |
| Diabetes, n (%) | 45 (16.5) |
| Hypothyroidism, n (%) | 31 (11.4) |
| TBI, n (%) | 66 (24.2) |
| Cerebrovascular disease, n (%) | 35 (12.8) |
| Cardiovascular disease, n (%) | 56 (20.5) |
| Mood disorder, n (%) | 125 (45.8) |
| Insomnia, n (%) | 55 (20.1) |
| OSA, n (%) | 47 (17.2) |
| Any neurological disorders, n (%) | 56 (20.5) |
| Psychiatric diseases except for mood disorder, n (%) | 54 (19.8) |

**Abbreviations:** Q1=the first quartile; Q3=the third quartile; SD=standard deviation; MoCA=the Montreal Cognitive Assessment; PROMPT= the PROspective Registry of Persons with Memory SyMPToms (PROMPT). **Variable pre-processing**: Any neurological disorders included the following: Parkinsonism, PD, multiple sclerosis, seizure/epilepsy, ALS, and history of delirium. Psychiatric diseases (except for depression, anxiety disorder, and mood disorder) included ADD/ADHD, bipolar disorder, OCD, panic disorder, PTSD, schizophrenia, psychotic disorder, or other neuropsychiatric symptoms (apathy, mood changes, worry, agitation, impulsivity, personality change, social inappropriateness). Smoked included current and former smokers. Any neurological signs included gait disorder, signs of frontal dysfunction, parkinsonism, motor neuron signs, neuro-opthalmologic signs, and focal or lateralizing signs. Used education category to impute missing years of education. Cardiovascular disease was defined if patient checked at least one of the following: coronary artery disease including MI, Atrial fibrillation or flutter, congestive heart disease, Pacemaker, PCI (Angioplasty), Coronary artery bypass graft (CABG), or Valve replacement/repair. Cerebrovascular disease was defined if patient checked at least one of the following: Ischemic stroke, Intracerebral hemorrhage, Unspecified stroke, TIA, or Endarterectomy/Carotid stent procedure. Mood disorder was defined if patient checked at least one of the following: depression, generalized anxiety disorder**,** mood disorder**,** or the use of anti-depressant medications. Hypertension was defined if hypertension was checked as one of the vascular risk factors, or one of the following cardiovascular medications was checked including ace-inhibitor, angiotensin receptor blocker, calcium-channel blocker, beta blocker, or diuretic. Dyslipidemia was defined if Dyslipidemia was checked as one of the vascular risk factors, or the cardiovascular medication was checked including Statin. Diabetes was defined if Type I diabetes or Type 2 diabetes was checked as one of the vascular risk factors. TBI was defined if chronic traumatic encephalopathy or previous concussions/Acute TBI was checked. Marital status was re-grouped as married/common-law vs the rest, the rest group include Widowed, Separated, Divorced, or Single, never married. Living arrangement was regrouped as with spouse/partner only vs the rest, the rest include alone, with spouse/partner and other(s), with child (not spouse/partner), with parent(s) or guardian(s), with sibling(s), with other relatives, or with non-relatives. Residence type was regrouped as single-family residence vs the rest, the rest include Retirement community assisted living/board and care home, skilled nursing facility/long-term care, or other.

**Table S2.** Variable measures in NACC (version 3)

| **Variables** | **N= 967** |
| --- | --- |
| **Outcome variables** |  |
| Dementia diagnosis in 3 years, n (%) | 224 (23.2) |
| Event time in months, median (Q1-Q3) | 17.9 (12.8-27.1) |
| **Potential predictors** |  |
| Age, mean (SD) | 72.6 (7.4) |
| Female, n (%) | 484 (50.1) |
| White, n (%) | 776 (80.2) |
| Education in years, mean (SD) | 16.1 (2.9) |
| Married, n (%) | 676 (69.9) |
| Family history, n (%) | 505 (52.2) |
| Right-handed, n (%) | 846 (87.5) |
| English first language, n (%) | 906 (93.7) |
| Years from initial decline^1^, mean (SD) | 3.0 (3.2) |
| Behavioral decline^2^, n (%) | 442 (45.7) |
| Motor function decline, n (%) | 155 (16.0) |
| Gradual function decline, n (%) | 776 (80.2) |
| Referred by health professionals, n (%) | 589 (60.9) |
| Primary reason for coming to ADC: research, n (%) | 693 (71.7) |
| GDS, mean (SD) | 2.3 (2.4) |
| MoCA, mean (SD) | 22.4 (3.4) |
| NPS total, mean (SD) | 1.8 (2.1) |
| Memory complaints (subjects or informants), n (%) | 881 (91.1) |
| Never Smoked, n (%) | 581 (60.1) |
| Body mass index, mean (SD) | 27.1 (4.9) |
| Hypertension, n (%) | 625 (64.6) |
| Diabetes, n (%) | 157 (16.2) |
| Hypercholesterolemia, n (%) | 603 (62.4) |
| Thyroid disease, n (%) | 176 (18.2) |
| Arthritis, n (%) | 528 (54.6) |
| Urinary incontinence, n (%) | 163 (16.9) |
| Cerebrovascular disease, n (%) | 83 (8.6) |
| Cardiovascular disease, n (%) | 238 (24.6) |
| TBI, n (%) | 184 (19.0) |
| Cancer, n (%) | 187 (19.3) |
| Use of nonsteroidal anti-inflammatory medication, n (%) | 504 (52.1) |
| Mood disorder, n (%) | 520 (53.8) |
| Sleep apnea, n (%) | 239 (24.7) |
| REM sleep behaviour disorder, n (%) | 63 (6.5) |
| Insomnia, n (%) | 159 (16.4) |
| PD signs, n (%) | 129 (13.3) |
| Vision, n (%) | 211 (21.8) |
| Hearing, n (%) | 742 (76.7) |
| B12 deficiency, n (%) | 90 (9.3) |

**Abbreviations:** Q1=the first quartile; Q3=the third quartile; SD=standard deviation; MoCA=the Montreal Cognitive Assessment; ^1^years from initial decline was defined as year differences between baseline line and the onset age of cognitive decline (based on clinician’s assessment, at what age did the cognitive decline begin). ^2^based on clinician’s judgement, is the subject currently experiencing any kind of behavioral symptoms?

**Figure S1.** The Identification of the PROMPT cohort with linked administrative health data

**Notes:** N=the number of individuals; PROMPT= the PROspective Registry of Persons with Memory SyMPToms (PROMPT); MCI=mild cognitive impairment; DAD=Discharge abstract database; Claims=practitioner claims; PIN=Pharmaceutical information network.

**Figure S2.** Identification of the NACC cohort.

Notes: N=the number of individuals; NACC= the National Alzheimer’s Coordinating Center; MCI=mild cognitive impairment; MoCA=the Montreal Cognitive Assessment; MMSE=Mini-mental State Examination.

**Table S3**. Model performance on the real-data assessed from 3-fold CV using Harrell’s c-index, integrated brier score (IBS), using mean and SD

|  | **PROMPT Sample** | | | **NACC Sample** | | |
| --- | --- | --- | --- | --- | --- | --- |
|  | **C-index** | **IBS** |  | **C-index** | **IBS** |  |
|  | | | | | | |
| **Cox** | **0.62** | **0.19** |  | **0.72** | **0.13** |  |
| Ridge-Cox | 0.62 | 0.20 |  | 0.73 | 0.14 |  |
| Lasso-Cox | 0.64 | 0.18 |  | 0.73 | 0.16 |  |
| EN-Cox | 0.65 | 0.19 |  | 0.73 | 0.15 |  |
| SurvTree | 0.54 | 0.35 |  | 0.68 | 0.23 |  |
| **RSF** | **0.62** | **0.20** |  | **0.73** | **0.13** |  |
| SSVM | 0.63 | 0.34 |  | 0.71 | 0.21 |  |
| SNN | 0.54 | 0.20 |  | 0.67 | 0.14 |  |
| **XGBoost** | **0.63** | **0.21** |  | **0.73** | **0.14** |  |

Abbreviations: SurvTree=Survival Tree; RSF= Random survival forests; SSVM=Survival support vector machine; SNN= Survival neural network; XGBoost=Extreme gradient boosting.

Table S4. Hyperparameter tuning search space and tuned results for PROMPT and NACC sample

| **Survival Models** | **Search space** | **Tuned results**  **For PROMPT** | **Tuned results**  **For NACC** |
| --- | --- | --- | --- |
| Ridge-Cox | $\lambda=\left( {10}^{-4}, {10}^{2} \right)$ | $\lambda=0.0002$ | $\lambda=0.566$ |
| LASSO-Cox | $\lambda=\left( {10}^{-4}, {10}^{2} \right)$ | $\lambda=0.064$ | $\lambda=0.016$ |
| ElasticNet-Cox | $\alpha=\left( 0,1 \right)$  $\lambda=\left( {10}^{-4}, {10}^{2} \right)$ | $\alpha=0.818$  $\lambda=0.173$ | $\alpha=0.009$  $\lambda=2.576$ |
| Survival tree | $cp =(0.001, 0.1)$    $minsplit =(1, 20)$ | $cp =0.016$  $minsplit =10$ | $cp =0.009$  $minsplit =16$ |
| Random Survival Forests | $mtry=(2, p-2)$  $min.node size =(1,20)$ | $mtry=2$  $min.node size =14$ | $mtry=37$  $min.node size =2$ |
| survival support vector machines | Gamma.mu = (2^-5^, 2 ^15^)  Kernel =c("linear kernel", "RBF kernel")  If RBF kernel, train kernel parameter: (2^-15^, 2^3^) | Gamma.mu=0.074  Kernel = linear kernel | Gamma.mu=0.358  Kernel = RBF kernel  RBF kernel parameter= 7.16 |
| Survival neural network | optimizer =”adam”,  activation = “relu",  dropout = (0, 0.5),  weight decay = (0, 0.5),  learning rate = (0, 0.5),  nodes = (2^3^, 2^10^),  k = (1, 10)  ##assume the same number of nodes per layer | dropout = 0.411,  weight_decay = 0.394,  learning_rate = 0.311,  nodes = 10,  k = 1 | dropout = 0.086,  weight_decay = 0.079,  learning_rate = 0.161,  nodes = 9,  k = 10 |
| Extreme gradient boosting | nrounds = (100,1000) with budget function,  eta = (0.01, 0.1),  subsample = (0.5, 0.9),  max depth = (3,10),  min child weight = (1,10),  column sample by tree = (0.5, 0.9)) | nrounds = 103,  eta = 0.015,  subsample =0.580,  max_depth = 9,  min_child_weight = 8,  colsample_bytree = 0.751 | nrounds = 143,  eta = 0.018,  subsample =0.815,  max_depth = 3,  min_child_weight = 5,  colsample_bytree= 0.809 |

Notes: *minsplit* refers to the minimum number of observations that must exist in a node in order for a split to be attempted. *cp*=complexity parameter. Any split that does not decrease the overall lack of fit by a factor of *cp* is not attempted. The main role of this parameter is to save computing time by pruning off splits that are obviously not worthwhile. Ceiling functions were used for mtry, which is number of the variables to possibly split in each node. p = the number of predictors (features). Minsplit or min.node.size, are the minimum number of observations that must exist in a node in order for a split to be attempted.
